# Effect of chlorpyrifos on sperm characteristics and testicular tissue changes in adult male rats

**Published:** 2017-12-15

**Authors:** Mojtaba Babazadeh, Gholamreza Najafi

**Affiliations:** 1 *Graduate Student, Faculty of Veterinary Medicine, Urmia University, Urmia, Iran;*; 2 *Department of Basic Sciences, Faculty of Veterinary Medicine, Urmia University, Urmia, Iran.*

**Keywords:** Chlorpyrifos, Histology, Rat, Sperm, Testis

## Abstract

Chlorpyrifos (CPF) is a broad spectrum organophosphate pesticide used for agricultural health purposes. Its principal mechanism of toxicity is the inhibition of acetylcholinesterase. The purpose of present study was to investigate the effects of CPF on testicular tissue and sperm parameters in male rats. Thirty-two healthy male rats were divided into two groups: a CPF-exposed group and a control-sham group. Control-sham group received corn oil (0.20 mL per day). The CPF was administered orally to male rats at 37 mg kg^-1^ BW for 45 days to evaluate the reproductive toxicity. In all rats, sampling for histological and sperm analyses was performed on days 5, 15, 30 and 45. The CPF caused a significant (*p *< 0.05) decrease in sperm count, viability and motility and increased immature sperms and DNA damage in sperm cells. Light microscopic analyses revealed increased arrested spermatogenesis, negative tubular differentiation and repopulation indexes and decreased Leydig cells number. These findings indicate that CPF has adverse effects on sperm cells and reproductive system of male rats.

## Introduction

The use of pesticides in the last fifty years due to agricultural intensification increased quickly in order to obtain higher yields. Chemicals cause irreparable damages to the environment as well as long-term health problems in humans and animals.^1 ^Pesticides as man-made chemicals designed to kill living organisms are biologically active materials including organophosphate (OP) compounds.^1^^,^^2^ The OP compounds are various groups of chemicals used in both domestic and industrial settings. Instances of OPs are insecticides such as diazinon, malathion, parathion, fenthion and chlorpyrifos (CPF). In farm workers, chronic occupational exposure to OP insecticides has been linked to neuropsychological effects in some studies. These effects have included difficulties in executive functions, psychomotor speed, memory, verbal, attention, processing speed and visual-spatial functioning.^3^ The CPF with formula 0, 0 -diethyl 0- 3, 5, 6-trichloro-2-pyridyl thiophosphonate is an OP pesticide used for agricultural and public health purposes.^4^ Principal mechanism of OP toxicity is inactivation of acetylcholine-esterase (AChE) through phosphorylating of the serine hydroxyl residue on AChE.^5^

Meanwhile, the induction of oxidative stress has also been indicated in CPF toxicity. ^6^ Recent investigations have demonstrated significant associations between maternal and paternal exposures to CPF and testicular damages. The CPF also brought about marked reduction in epididymal and testicular sperm counts and a decrease in serum testosterone concentration in exposed males rats. Further, histopathological examinations of testes showed mild to severe degenerative changes in seminiferous tubules at various dose levels of CPF.^7^

Therefore, the aim of present study was to investigate the effects of CPF on testicular tissue and examine the effects of this chemical compound on quantity, quality and morphology of epididymal sperms in chronic exposure in mature male rats.

## Materials and Methods

**Chemicals. **The CPF 40.80% (Trust Chem Co.,Ltd, Nanjing, China) was purchased from agricultural chemical store. The oral LD50 for CPF in rats is 270 mg kg^-1^. 

**Animals and treatments. **All conducted experimental protocols were approved by the ethical committee of Urmia University based on proofed principles for laboratory animal care (P 3.155). In order to perform current study, 32 male Wistar rats weighing between 185 to 203 g were obtained from the Animal Resource Center of the Faculty of Veterinary Medicine, Urmia University, Urmia, Iran. The rats were acclimatized for a one week and the diet and water were given *ad libitum*. All stress factors were diminished into minimum and a standard condition (12 hr light-dark cycle, temperature 25 ± 2 ˚C, humidity 50 ± 10%) was prepared. After acclimation period, all animals were randomly divided into two groups containing 16 rats each. The control-sham groups received 0.20 mL corn oil (Sunar, Adana, Turkey) and in CPF groups, CPF was administrated at a dose of 37 mg kg^-1^ body weight orally. Sampling for histological and sperm analyses was performed on days 5, 15, 30 and 45.

**Testicular weight determination. **Following induction of anesthesia with intraperitoneal injection of ketamine (75 mg kg^-1^; Alfasan, Woerden, The Netherlands), on days 5, 15, 30 and 45 all rats in each group were euthanized by dislocation of cervical vertebrae and immediately following weighting of total body weight, the testes were quickly excised free of surrounding connective tissues and weighed on a scale (Delta Range, Tokyo, Japan).

**Histopathological analyses. **All tissue samples were fixed in 10% saline formalin solution fixative and subsequently, dehydrated in ethyl alcohol and cleared in xylene for histological investigations. Sections (7 μm thickness) were stained with hematoxylin and eosin and iron-weigert for histopathological and morphometric assessments. All samples were studied by multiple magnifications (400× and 1000×). For quantification of cells and their dimensions, a 100 μm morphometrical lens-device was used. To measure germinal epithelium height (GEH) and seminiferous tubules diameter (STD), 200 round or nearly-round cross-sections of the seminiferous tubules were randomly chosen from each rat. Using an ocular micrometer with light microscopy, the GEH at four equidistant points of each seminiferous tubule cross-section was measured and the mean was recorded. The STDs were measured across the minor and major axes and their means were obtained.^8^ To evaluate the mononuclear immune and Leydig cells, the number of these cells was counted in one mm^2^ of the interstitial connective tissue by using morphometrical lens-device (Olympus Co., Düsseldorf, Germany). 

**Tubular differentiation index (TDI). **To estimate TDI, the percentage of seminiferous tubules with more than three layers of differentiated germinal cells from spermatogonia type A were counted and considered to be TDI positive.^8^

**Repopulation index (RI) calculation. **To determine RI, the ratio of active spermatogonia (spermatogonia type B with dark nuclei as seen by iron-weigert staining) to inactive spermatogonia (spermatogonia type A with light nuclei as seen by iron-weigert staining) in seminiferous tubules was calculated in prepared sections.^9^

**Spermiation index (SPI). **The SPI is the percentage of seminiferous tubules with normal spermiation. Semini-ferous tubules with normal spermiation were considered to be SPI positive.^10^

**Epididymal sperms analysis. **Epididymal tissues were dissected out carefully under a 10 time magnification provided by stereo zoom microscope (model TL2; Olympus Co., Tokyo, Japan). Then, the both epididymides (right and left) were divided into three segments; caput, corpus and cauda. The caudal epididymal sperms were collected by slicing the caudal part of epididymis in to small pieces in 10 mL medium for 1-cell rat embryos (mR1ECM) for 30 min, in an atomosphere of 5% CO_2_ at 37 ˚C in culture device (Leec Ltd., Nottingham, UK).^11^

**Preparation of mR1ECM. **To prepare the stock A, 0.239 g potassium chloride (Sigma, St. Louis, USA), 6.420 g sodium chloride (Sigma), 1.352 g glucose (Sigma), 0.075 g penicillin G (Sigma), 0.050 g streptomycin (Sigma), 1.90 mL sodium lactate (Sigma) were dissolved in 100 mL distilled water and the solution was sterilized by filtration through 0.22 µm filter and stored at 4 ˚C. To prepare the stock B, the reagents including 0.102 g magnesium chloride (Sigma) and 0.294 g calcium chloride (Sigma) were dissolved in distilled water and adjusted to the volume of 100 mL. The solution was sterilized by filtration through 0.22 µm filter and stored at 4 ˚C. All reagents (10 mL Stock A, 10 mL Stock B, 0.210 g sodium bicarbonate (Sigma), 0.005 g sodium pyruvate (Sigma), 0.014 g L-glutamine (Sigma), 2 mL essential amino acids and 1 mL non-essential amino acids) were dissolved in distilled water and adjusted to the volume of 100 mL. The osmotic pressure was adjusted to about 244-246 mOsm. The solution was sterilized by filtration through 0.22 µm filter and stored at 4 ˚C.^12^

**Sperm count. **The epididymal sperm count was determined by the standard hemocytometeric method. After dilution of epididymal sperm (1:20) in distilled water, 10 µL of this diluted specimen were transferred to each of the counting chambers of the hemocytometer (HBG, Hamburg, Germany) which was allowed to stand for 5 min in a humid chamber to prevent drying. The cells were sedimentated during this time counted with a light microscope at 400×. The sperm count was expressed as a number of sperm per milliliter.^13^

**Sperm viability.** To determine the percentage of sperm viability, the 20 μL of epididymal sperm suspension were mixed with 20 μL of 0.05% eosin-Y in a sterile test tube. After 20 sec, 50 μL of nigrosin were added and mixed thoroughly. The mixture of stained sperm was smeared on the slide and examined under bright field microscope (Olympus) at 400×. A total of 200 sperm cells were examined on each slide. Sperms with altered plasma membrane seemed to be pink and sperms with intact plasma membrane were not stained. Finally, the results were expressed in percentage.^14^

**Sperm motility. **The percentage of sperm motility was evaluated visually by a light microscope at 400× magnification. For this procedure, one drop of sperm suspension was placed on a slide and a cover slip was placed over the droplet. At least 10 fields were observed and motile sperms percentages were calculated.^14^

**Aniline blue staining. **The aniline blue staining was performed in order to evaluate sperm chromatin condensation. The 5 μL of the prepared spermatozoa were smeared and allowed to dry in laboratory temperature. Then, the prepared smears were fixed in 3.00% buffered glutaraldehyde (Sigma) in 0.20 M phosphate buffer (pH: 7.20) for 30 min. After that, the slides were stained with 5.00% aqueous aniline blue mixed with 4.00% acetic acid (Merck, Darmstadt, Germany) (pH: 3.50) for 5 min and then 100 sperm cells per slide were analyzed and the percentage of unstained sperm heads (representing condensed chromatin) was calculated and compared between groups.^15^

**Assessment of sperm DNA damage**. Acridine orange (AO; Sigma) test is a sperm chromatin structure microscopic assay which reflects sperm chromatin denaturation. A small aliquot of the sperm suspension was spread on the glass slides. The slides were then air dried and ﬁxed in Carnoy’s solution (methanol/acetic acid, 3:1, Sigma) for 2 hr. The slides were then stained for 5 min with freshly prepared AO solution (19.00%) in phosphate citrate (Sigma) dye. After washing with deionized water, the slides were examined using a ﬂuorescent microscope (Leitz, Wetzlar, Germany; excitation of 450 to 490 nm). Two hundred sperms were evaluated with a fluorescence microscope and sperm heads with intact chromatin had green fluorescence, while those with denatured chromatin had orange-red staining.^16^

**Statistical analyses. **Results were analyzed using SPSS (version 21.0; SPSS Inc., Chicago, USA). The comparisons between groups were made by analysis of variance (One-Way ANOVA) followed by Bonferroni post-hoc test. All data were expressed as the mean ± standard error of mean (SEM). The statistical significance level was set at *p *< 0.05.

## Results

**Testicular weight and body weight. **Both CPF and corn oil treatments had no effect on food and water consumptions. Corn oil did not exert any significant effect on body weight in the control-sham group, while administration of CPF diminished body weight in the CPF groups. The CPF exposure caused a significant decrease in rats body weight on days 30 and 45 compared to control- sham and CPF group on day 5 (*p *< 0.05). Testicular weight decreased significantly in CPF-administrated rats on day 45 compared to control-sham and CPF groups on days 5, 15 and 30 (*p *< 0.05; Table 1).

**Fig. 1:**
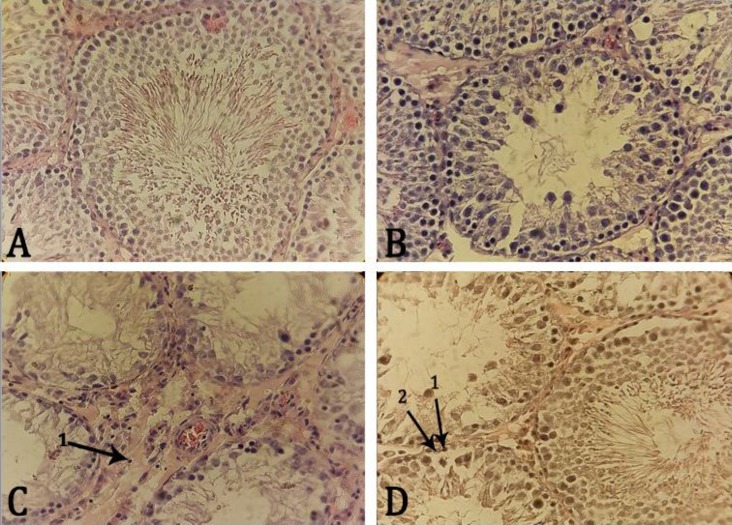
Photomicrograph of rat testis sections: A) control-sham group, seminiferous tubules with no histological changes in germinal cell proportion and no edema in the interstitial tissue are present. The spermatogenesis and spermiogenesis processes are normal and seminiferous tubules with a high proportion of spermatozoa are observed. (H & E, 400×), B) The CPF group on day 15, edema in interstitial connective tissue and irregular arrangement and detachment of spermatogenic cells are observed in some areas. (H & E, 400×), C) The CPF group on day 45, showing severe germ cell loss and vacuolization, sloughing of germ cells into the tubular lumen and impaired spermatogenesis; seminiferous tubules with negative tubular differentiation and repopulation indices as well as severe edema and infiltration of immune mononuclear cells (1) in interstitial connective tissue can be seen. (H & E, 400×) and D) The CPF group on day 30, exhibiting interstitial connective tissue edema, partially sloughing of germ cells into the tubular lumen and negative repopulation index. Spermatogonium type B (1), Spermatogonium type A (2). Iron-weigert staining technique (400×).

**Immune mononuclear and Leydig cells number.** The interstitial connective tissue infiltration of the immune mononuclear cells was elevated in the CPF groups in comparison with control-sham group. Immune mono-nuclear cells increased significantly in CPF groups on days 30 and 45 compared to control-sham and CPF groups on days 5, 15 and 30 (*p *< 0.05). Leydig cells showed severe hypertrophy and cytoplasmic granulation in all CPF groups as well as significant decrease in CPF groups on days 30 and 45 compared to control-sham and CPF groups on days 5 and 15 (*p *< 0.05). There was no significant difference in Leydig cells number between CPF groups on days 30 and 45 (*p *> 0.05), (Fig. 1 and Table 2). 

**Table 1:** Effect of chlorpyrifos (CPF) on body weight and testicular weight. Values are expressed as mean ± SEM.

**Groups**	**Body weight (g)**	**Testicular weight (g)**
***5 days***		
**Control**	194.00 ± 4.92^a^	0.90 ± 0.03 ^a^
**CPF**	195.00 ± 2.17 ^a^	0.92 ± 0.02 ^a^
***15 days***		
**Control**	195.90 ± 0.34 ^a^	0.91 ± 0.02 ^a^
**CPF**	191.33 ± 2.75 ^a^	0.89 ± 0.01 ^a^
***30 days***		
**Control**	195.48 ± 5.52 ^a^	0.88 ± 0.02 ^a^
**CPF**	170.67 ± 1.75^b^	0.84 ± 0.03 ^a^
***45 days***		
**Control**	194.18 ± 3.58 ^a^	0.88 ± 0.00 ^a^
**CPF**	170.00 ± 3.50^b^^c^	0.70 ± 0.02^b^

abc Different superscript letters in each column indicate significant differences between all groups (*p *< 0.05).

**Table 2:** Effects of chlorpyrifos (CPF) on number of immune mononuclear cells (IMC) and Leydig cells (LC) in each mm^2^ of testicular interstitial connective tissue. Values are expressed as mean ± SEM.

**Groups**	**LC (No. per mm** ^2^ **)**	**IMC (No. per mm** ^2^ **)**
***5 days***		
**Control**	7.33 ± 0.43^a^	6.67 ± 0.60 ^a^
**CPF**	7.33 ± 0.60 ^a^	8.33 ± 0.60 ^a^
***15 days***		
**Control**	7.42 ± 0.25 ^a^	6.86 ± 0.14 ^a^
**CPF**	9.67 ± 1.32 ^a^	6.67 ± 0.43 ^a^
***30 days***		
**Control**	7.56 ± 0.33 ^a^	6.15 ± 0.14 ^a^
**CPF**	3.33 ± 0.16^b^	15.67 ± 0.72^b^
***45 days***		
**Control**	7.96 ± 0.09 ^a^	6.69 ± 0.32 ^a^
**CPF**	3.67 ± 0.16^b^	15.50 ± 0.61^b^

abc Different superscript letters in each column indicate significant differences between all groups (*p *<0.05).

**Fig. 2:**
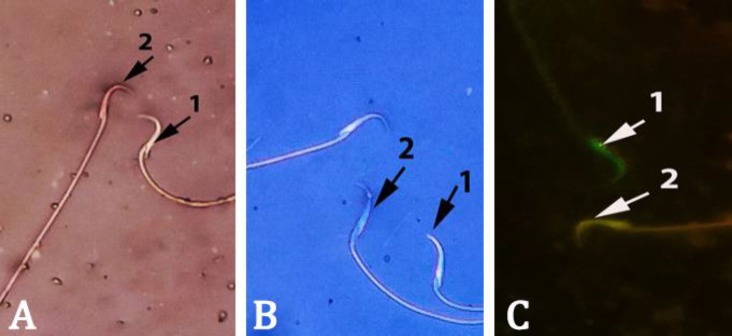
Rat spermatozoa stained with A) Eosin-nigrosin, viable sperm (1) remained colorless while non-viable sperm (2) stained red, B) Aniline-blue, mature sperm (1) with discolored protamine and immature sperm (2) characterized by nuclear histone proteins stained dark-blue and C) Acridine orange, green spermatozoa with double-stranded DNA (1) and yellow spermatozoa with single-stranded DNA (2). (1000×).

**Testicular capsule thickness (TCT), germinal epithelium height and seminiferous tubules diameter. **As shown in Table 3, TCT was similar between control-sham groups and CPF groups. Compared with the control-sham group, GEH decreased significantly in CPF group on day 45 (*p *< 0.05). There was no significant difference in GEH between CPF groups on days 5, 15 and 30 and control-sham groups (*p *> 0.05). There was a reduction in STD on day 45 after CPF administration. There was no significant difference in STD between all groups (*p *> 0.05), (Fig. 1 and Table 3).

**Tubule differentiation index, spermiation index and repopulation index.** The CPF caused vacuolization in the seminiferous tubules. Also, seminiferous tubules revealed loss of spermatogenic cells. The CPF significantly reduced TDI on day 45 compared to control-sham and CPF groups on days 5, 15 and 30 (*p* < 0.05). In the CPF groups, SPI and RI in days 30 and 45 of experiment were significantly decreased in comparison with control-sham and CPF groups on days 5 and 15 (*p* < 0.05), (Fig. 1 and Table 3).

**Table 3:** Testicular capsule thickness, germinal epithelium height, seminiferous tubules diameter, tubule differentiation index, spermiation index and repopulation index in all experimental groups. Values are expressed as mean ± SEM.‎

**Groups**	**TCT (µm)**	**GEH (µm)**	**STD (µm)**	**TDI (%)**	**SPI (%)**	**RI (%)**
***5 days***						
**Control**	35.67 ± 1.04 ^a^	60.33 ± 1.52 ^a^	190.33 ± 2.36^a^	98.74 ± 0.59 ^a^	85.85 ± 1.20 ^a^	94.67 ± 0.79 ^a^
**CPF**	33.00 ± 1.80 ^a^	57.00 ± 1.52 ^a^	191.00 ± 1.80 ^a^	98.17 ± 0.83 ^a^	85.03 ± 0.90 ^a^	91.16 ± 2.15 ^a^
***15 days***						
**Control**	34.50 ± 0.61 ^a^	60.75 ± 1.30 ^a^	190.61 ± 2.40 ^a^	99.08 ± 0.12 ^a^	89.66 ± 0.49 ^a^	93.84 ± 1.08 ^a^
**CPF**	34.00 ± 1.32 ^a^	57.67 ± 1.89 ^a^	186.33 ± 6.65 ^a^	97.56 ± 1.11 ^a^	82.83 ± 1.16^ab^	89.00 ± 0.95 ^a^
***30 days***						
**Control**	33.37 ± 2.29 ^a^	60.74 ± 2.93 ^a^	191.75 ± 4.24 ^a^	98.95 ± 0.16 ^a^	88.19 ± 1.85 ^a^	91.49 ± 1.56 ^a^
**CPF**	32.67 ± 0.76 ^a^	55.67 ± 1.89 ^a^	190.33 ± 6.44 ^a^	97.06 ± 0.93 ^a^	73.72 ± 1.39^abc^	76.87 ± 0.76^b^
***45 days***						
**Control**	34.90 ± 1.81 ^a^	61.00 ± 1.91 ^a^	192.95 ± 5.61 ^a^	98.98 ± 0.19 ^a^	88.77 ± 3.34 ^a^	93.60 ± 2.18 ^a^
**CPF**	34.33 ± 1.04 ^a^	36.00 ± 5.56 ^b^	184.00 ± 4.76^a^	90.35 ± 0.97 ^b^	71.02 ± 1.15^cd^	66.73 ± 1.20^c^

CPF: Chlorpyrifos; TCT: Testicular capsule thickness; GEH: Germinal epithelium height; STD: Seminiferous tubules diameter; TDI: Tubule differentiation index; SPI; Spermiation index; RI: Repopulation index.

abc Different superscript letters in each column indicate significant differences between all groups (*p *< 0.05).

**Table 4:** Effect of chlorpyrifos (CPF) on epididymal sperm parameters. Values are expressed as mean ± SEM.‎

**Groups**	**Sperm count (10** ^6^ ** ml** ^-1^ **)**	**Sperm viability (%)**	**Sperm motility (%)**	**Immature sperm (%)**	** DNA damage ** **(%)**	**Sperm count (10** ^6^ ** ml** ^-1^ **)**
***5 days***						
**Control**	74.67 ± 1.60^a^	71.00 ± 2.64 ^a^	71.68 ± 2.57 ^a^	10.67 ± 0.76 ^a^	8.29 ± 0.47 ^a^	74.67 ± 1.60 ^a^
**CPF**	73.00 ± 2.00 ^a^	57.33 ± 2.25 ^a^	57.06 ± 3.06 ^a^	11.00 ± 0.86 ^a^	11.15 ± 0.95 ^a^	73.00 ± 2.00 ^a^
***15 days***						
**Control**	73.29 ± 2.06 ^a^	70.83 ± 2.37 ^a^	70.90 ± 1.50 ^a^	10.42 ± 0.99 ^a^	7.98 ± 1.36 ^a^	73.29 ± 2.06 ^a^
**CPF**	68.67 ± 6.82 ^a^	56.67 ± 3.32 ^a^	54.82 ± 2.14 ^a^	20.00 ± 1.80 ^b^	13.52 ± 0.81 ^b^	68.67 ± 6.82 ^a^
***30 days***						
**Control**	67.99 ± 1.59 ^a^	72.58 ± 4.19 ^a^	69.96 ± 3.67 ^a^	10.22 ± 0.52 ^a^	8.07 ± 1.27 ^a^	67.99 ± 1.59 ^a^
**CPF**	36.33 ± 2.56^b^	42.00 ± 1.32^b^	42.42 ± 3.80^b^	31.42 ± 2.50^c^	20.09 ± 0.94^bc^	36.33 ± 2.56^b^
***45 days***						
**Control**	73.19 ± 1.69 ^a^	73.99 ± 2.71 ^a^	75.21 ± 2.54^a^	10.30 ± 0.77^a^	8.92 ± 1.19 ^a^	73.19 ± 1.69 ^a^
**CPF**	31.00 ± 1.50^bc^	31.33 ± 3.01^bc^	29.55 ± 7.36^bc^	38.00 ± 1.80^cd^	25.97 ± 2.59^bcd^	31.00 ± 1.50^bc^

abc Different superscript letters in each column indicate significant differences between all groups (*p *< 0.05).

**Sperm parameters. **The CPF on days 30 and 45, caused a significant decrease in the epididymal sperm concentration, viability (Fig. 2A) and motility compared to control-sham and CPF groups on days 5 and 15 (*p *< 0.05). There was no significance different between CPF groups on days 30 and 45 in above-mentioned parameters (*p *> 0.05), (Table 4). As shown in Table 4, the percentages of immature sperms increased significantly in CPF groups on days 15, 30 and 45 compared to control-sham group (*p *< 0.05), (Fig. 2B). The results of AO staining revealed that the percentages of sperms with DNA damage in CPF-exposed animals on days 15, 30 and 45 were significantly higher than control-sham rats (*p *< 0.05). Also, there was a significant increase in sperm damage between CPF groups on days 15 and 45 (*p *< 0.05), (Fig. 2C). 

## Discussion

Different OP insecticides are in wide use worldwide and 5.00% of the world’s populations are directly exposed to these insecticides. According to recent reports, this population is calculated to be 2.60 million persons.^17^ The OP pesticides are fat-soluble macro-molecular substances that can be absorbed through the lungs, skin and gastrointestinal tract and bind to red blood cell AChE. The OPs inactivate AChE by phosphorylating the serine hydroxyl group located at the active site of AChE.^18^^-^^20^

The effects of CPF on testicular and body weights were assessed in pregnant CF-1 mice following acute duration oral exposure at doses as high as 25 mg kg^-1^ per day CPF in cottonseed oil.^21^ A statistically significant decrease in mean body weight gain was observed in animals exposed to 25 mg kg^-1 ^per day CPF. Similar effects have been observed in rats.^22^^,^^23^ A single dose of 100 mg kg^-1^ CPF administered via gavage in corn oil caused a 13.30% decrease in the body weight of male rats after 24 hr. Decreased body weight was not seen at doses of 50 mg kg^-1^ or less.^23^

Similarly, pregnant rats exposed via gavage (15 mg kg^-1^ per day in corn oil) experienced a statistically significant decrease in mean body weight gain.^22^^,^^24^ In this study, the body and testicular weights in CPF-administered rats were significantly lower than those of the control-sham groups. This could be attributed to severe parenchyma atrophy in the seminiferous tubules following CPF treatment. Also, spermatogenic arrest and inhibition of steroid biosynthesis of Leydig cells, may contribute to the decline of testicular weight.^25^ Chronic exposure to CPF elicits numbers of other toxic effects including hepatic dysfunction, immunological abnormalities, genotoxicity, teratogenicity, neurotoxicity and neurobehaviourial changes.^26^^-^^29^ Some reports have indicated that human immune function may be altered by CPF exposure.^30^ Findings of the present study showed that CPF increases infiltration of immune mononuclear cells in the interstitial connective tissue in test groups. Moreover, it has been shown that CPF increases oxidative stress, apoptosis and DNA damage in the exposed organisms.^31^

It has been reported that CPF causes immunological abnormalities and induces oxidative stress and tissue damages. One possible mechanism may be the inhibition of mitochondrial ATP production through the uncoupling of oxidative phosphorylation leading to the generation of reactive oxygen species (ROS). The ROS may be involved in the toxicity of various pesticides.^32^^-^^36^ The ROS are the product of normal cellular metabolism. Sperm is a type of cell that manufactures free oxygen radicals. With reactive oxygen radical production at low levels, sperm cell capacitation, acrosomal reaction and sperm binding to the zona pellucida take place.^37^ Uncontrolled ROS production leads to sperm abnormalities, spermatogenic cells degeneration and infertility.^38^ In the present study, elevated abnormal sperms (sperms with DNA damage and immature sperms), degenerated germinal cells and decreased sperm motility and viability were observed in test groups.

Possibly, CPF may exert oxidative stress in the testicles of the test group rats and consequently increased sperm abnormality. It has been demonstrated that degenerative changes occur in the seminiferous tubules of rats received malathion.^39^ Our investigations on the effects of CPF on germinal epithelium height revealed that this parameter decreases in test group rats. There were sloughing and disorganization of spermatogenic cells with their exfoliation in seminiferous tubules lumen. Thus, the reduction in thickness of the somniferous tubules epithelium can be due to degenerative effects of the pesticides. Dimethoate, an OP pesticide, causes rarefaction of Leydig cells.^40^ Evaluation of Leydig cells distribution in the testicular interstitial tissue in this study revealed significant decrease in the population of these cells only after 30 and 45 days following CPF administration. The results of this study showed that CPF treatment results in a TDI positive percentage reduction on day 45. It should be noticed that when the TDI percentage decreases, it indicates that the epithelial layer is undergoing degeneration and thinning. The previous studies reported that dimethoate causes testicular damages characterized by moderate to severe seminiferous tubule degeneration, sloughing, atrophy and degeneration of germ cells and partial arrest of spermatogenesis.^41^ Recent researches revealed that dimethoate causes adverse effects on reproductive performance of male mice including sperm viability, motility and density impairment.^42^ Accordingly, it has been shown that bromopropane decreases spermatogenesis by adversely affecting spermatogonia followed by depletion of spermatocytes, spermatids and spermatozoa and subsequent testicular atrophy. Percentage of seminiferous tubules without sperms was increased in CPF-administrated groups. Dimethoate was also shown to be able to decrease sperm viability, motility and density.^43^ Also, we noticed that the RI decreases in CPF groups. This indicates that the ratio of spermatogonia type B to the spermatogonia type A reduces by CPF administration, thus CPF treatment can lead to spermatogonia type B population reduction. 

In conclusion, these data indicate that male reproductive tract can be considered as a target for CPF. The CPF administration can result in histological damages in testicular tissue, increased sperm mortality, immature sperms and sperm DNA damage and sperm count, motility and viability reduction. Hence, CPF may cause infertility following chronic exposure.
